# A low RENAL Nephrometry Score can avoid the need for the intraoperative insertion of a ureteral catheter in robot-assisted partial nephrectomy

**DOI:** 10.1186/s12957-021-02146-0

**Published:** 2021-02-04

**Authors:** Kenichi Nishimura, Yuichiro Sawada, Naoya Sugihara, Keisuke Funaki, Kanae Koyama, Terutaka Noda, Tetsuya Fukumoto, Noriyoshi Miura, Yuki Miyauchi, Tadahiko Kikugawa, Takashi Saika

**Affiliations:** Department of Urology, Ehime University Graduate school of Medicine, Shitsukawa, Toon, Matsuyama, Ehime 791-0295 Japan

**Keywords:** RAPN, RENAL Nephrometry Score, Collecting system entry

## Abstract

**Background:**

Intraoperative urinary collecting system entry (CSE) in robot-assisted partial nephrectomy (RAPN) may cause postoperative urinary leakage and extend the hospitalization. Therefore, identifying and firmly closing the entry sites are important for preventing postoperative urine leakage. In RAPN cases expected to require CSE, we insert a ureteral catheter and inject dye into the renal pelvis to identify the entry sites. We retrospectively analyzed the factors associated with intraoperative CSE in RAPN and explored the indications of intraoperative ureteral catheter indwelling in RAPN.

**Methods:**

Of 104 Japanese patients who underwent RAPN at our institution from August 2016 to March 2020, 101 were analyzed. The patients were classified into CSE and non-CSE groups. The patients’ background characteristics, RENAL Nephrometry Score (RNS), and surgical outcomes were analyzed.

**Results:**

Intraoperative CSE was observed in 41 patients (41%). The CSE group had a significantly longer operative time, console time, ischemic time, and hospital stay than the non-CSE group. In a multivariable analysis, the N-score (odds ratio [OR] = 3.9, *P* < 0.05) and RNS total score excluding the L-score (OR = 3.1, *P* < 0.05) were associated with CSE. In a logistic regression analysis, CSE showed a moderate correlation with the RNS total score excluding the L-score (AUC 0.848, cut-off 5, sensitivity 0.83, specificity 0.73).

**Conclusion:**

A ureteral catheter should not be placed in patients with an RNS total score (excluding the L-score) of ≤ 4.

## Introduction

Partial nephrectomy for renal cell carcinoma is a standard treatment that can be expected to preserve the renal function while maintaining cancer control, but it carries more complications than radical nephrectomy. Postoperative urine leakage, which is a complication unique to partial nephrectomy, occurs in 0.3–13.3% of cases [1, 2].

Once postoperative urine leakage is found, invasive treatment, such as placing a ureteral catheter or surgical intervention, is required for management. Postoperative urine leakage is caused mainly by insufficient closure of intraoperative collecting system entry (CSE) [3]. Therefore, identifying and firmly closing the entry sites are important to prevent postoperative urine leakage.

One way to identify entry sites is via visual recognition of dye in the renal pelvis with the insertion of a ureteral catheter during dissection of a renal tumor [4, 5]. However, placing a ureteral catheter prolongs the operative time and increases the expense [5]. Therefore, the pre-surgical prediction of CSE can avoid needless ureteral catheter placement.

We retrospectively analyzed the factors associated with intraoperative CSE in robot-assisted partial nephrectomy (RAPN) and explored the indications of intraoperative ureteral catheter indwelling in RAPN.

## Patients and methods

Our institutional review board-approved retrospectively maintained institutional database was queried to identify RAPN cases performed at our hospital from August 2016 to March 2020.

The patients were divided into a CSE group and non-CSE group, and the patient background characteristics (age, gender, height, weight, body mass index [BMI], tumor size), RENAL Nephrometry Score (RNS), and surgical outcomes (operative time, console time, ischemic time, discharge) were analyzed.

For variables with a non-normal distribution, data are presented as the median, and the groups were compared using Mann-Whitney’s *U* test. Categorical variables were compared using the chi-squared test. A comparative analysis was performed between the CSE and non-CSE groups. Univariable and multivariable logistic regression analyses were performed to calculate the odds ratios (ORs) for factors influencing CSE. A receiver operating characteristics (ROC) curve was used to analyze the utility of the RNS for predicting the risk of CSE in RAPN.

Significance was defined as *P* < 0.05. All analyses were performed using EZR.

### Surgical method

The surgical procedure for RAPN used in our institution is described. The method of approach (retroperitoneal, transperitoneal) is determined by the localization of the tumor. In cases where intraoperative CSE is expected, a ureteral catheter (Open End Ureteral Axxcess^TM^ Catheter, 6F, Boston Scientific) is inserted into the renal pelvis using a flexible cystoscope after the introduction of general anesthesia. During dissection of a renal tumor with renal artery clamping, a dye is injected into the renal pelvis to identify the entry sites of the collection system. The entry sites are then sutured using a 3-0 absorbent monofilament suture. Bleeding at the dissection surface is stopped using soft coagulation, and then the renal artery is de-clamped.

Usually, the renal parenchyma is not sutured, and the resection surface is covered with an absorbable fibrin sealant patch and fibrin glue. The ureteral catheter is removed before the patient is awakened from anesthesia in all cases. A drain is placed in all cases. The urethral catheter and drain are removed the day after surgery.

## Results

Of the 104 Japanese patients who underwent RAPN at our institution between August 2016 and March 2020, 3 were excluded from the analysis due to transition of total nephrectomy (1), open conversion (1), and two tumors in the same kidney (1), thus leaving 101 patients who could be evaluated.

The CSE group included 41 patients, and the non-CSE group included 60 patients. There were no significant differences in the background characteristics (age, height, weight, BMI, Charlson comorbidity index) between the two groups. The CSE group had a significantly longer operative time, console time, ischemic time, and hospital stay than the non-CSE group. Postoperative urine leakage occurred in 5 cases (5%), all in the CSE group. The entry sites were sutured. All cases recovered following the placement of a urethral catheter and ureteral stent after the detection of urinary leakage (Table 1). Significant differences were observed in the tumor anatomical factors, such as the tumor size, R-score, E-score, N-score, RNS total score, and RNS total score excluding the L-score (Tables 1 and 2).

**Table 1:** Patients’ characteristics

	CSE (*n* = 41)	non-CSE (*n* = 60)	*P value*
Age(yr)	70	68	n.s.
Gender			
Male	12(29%)	44(73%)	
Female	29(71%)	16(27%)	
Height(cm)	162	164	n.s.
Weight(kg)	64	65.5	n.s.
BMI(kg/m2)	25	23.9	n.s.
Side			n.s.
Left	18(44%)	25(42%)	
Right	23(56%)	35(58%)	
Charlson comorbidity index			n.s.
0-1	28(68%)	42(70%)	
>1	13(32%)	18(30%)	
Tumor size(mm)	34	23	<0.05
Clinical T stage			<0.05
T1a	25(61%)	55(92%)	
T1b	16(39%)	5(8%)	
Ureteral catheter placement	29(71%)	5(8%)	<0.05
Operation time(min)	183	154	<0.05
Consol time(min)	130	98.5	<0.05
Ischemic time(mim)	23	12	<0.05
Postoperative urine leakage	5(12%)	0	<0.05
Discharge(day)	9	7	<0.05

**Table 2:** RNS and CSE

	CSE (*n* = 41)	non-CSE (*n* = 60)	*P value*
**<R>adius**			<0.05
≦ 4 cm (1 point)	28(68%)	57(95%)	
> 4 to <7 cm (2 points)	13(32%)	3(5%)	
**<E>xophyic properties**			<0.05
≧ 50% (1 point)	14(34%)	37(62%)	
< 50%(2 points)	17(41%)	21(35%)	
Entirely (3 points)	10(25%)	2(3%)	
**<N>earness**			<0.05
≧ 7 mm (1 point)	6(15%)	41(68%)	
> 4 to < 7 mm (2 points)	3(7%)	10(17%)	
≦ 4 mm (3 points)	32(78%)	9(15%)	
**<L>ocation**			n.s.
Middle-kidney (1 point)	10(24%)	28(47%)	
Crosses polar line (2 points)	15(37%)	14(23%)	
Polar (3 points)	16(39%)	18(30%)	
**R.E.N.A.L score(median)**			
Total score	8	6	<0.05
Total score excluding L-score	6	4	<0.05

**Fig. 1:**
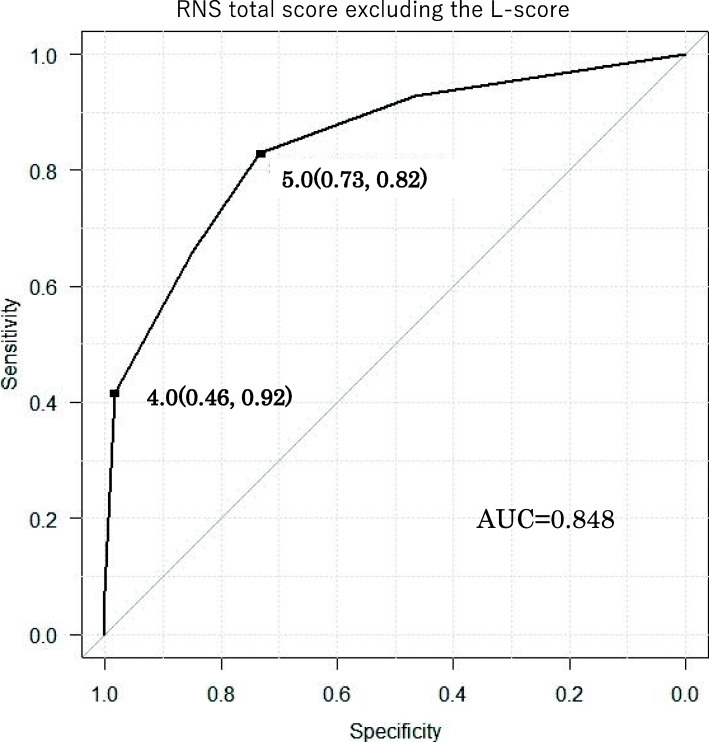
Receiver operating characteristics (ROC) curve: RNS total score excluding the L-score

In a multivariable analysis, the N-score (OR = 3.9, *P* < 0.05) and RNS total score excluding the L-score (OR = 3.1, *P* < 0.05) were associated with CSE (Table 3). In the logistic regression analysis, CSE showed a moderate correlation with the RNS total score excluding the L-score (AUC 0.848, cut-off 5, sensitivity 0.83, specificity 0.73) (Fig. 1).

**Table 3:** RNS and CSE in multivariable analyses

	OR	95%Cl	*P* value
<R>adius	4.2	0.9-19.8	n.s.
<E>xophyic properties	1.6	0.7-3.6	n.s.
<N>earness	3.9	2.1-7.5	<0,05
<L>ocation	1	0.5-2	n.s.
R.E.N.A.L score(median)			
RNS total score	0.9	0.5-1.8	n.s.
RNS total score excluding the L-score	3.1	1.4-7.2	<0.05

## Discussion

Factors associated with difficulty in partial nephrectomy are divided into tumor-related factors (tumor size and position) and patient-related factors (BMI, abdominal surgery history, etc.). The RNS is a score of anatomical factors of the tumor and consists of five components: <R>, <E>, <N>, <A>, and <L>. With a total score of 4–6 points being low, 7–9 points moderate, and 10–12 points high, it is considered that the higher the score, the longer the operative time and ischemic time of partial nephrectomy. The RNS is widely used because it can be easily evaluated using computed tomography or magnetic resonance imaging [6]. In our institution, the RNS has been used to evaluate preoperative difficulty, with a higher score considered to reflect a longer operative time and ischemic time, as in another report [7].

Zargar et al. analyzed a total of 1019 cases (452 RAPN cases and 567 laparoscopic partial nephrectomy (LPN) cases) for postoperative urine leakage. On a multivariable analysis, tumor proximity to the collecting system significantly increased the occurrence of postoperative urine leakage (OR = 9.2; *P* < 0.01) [5]. Mayer et al. analyzed the factors associated with CSE in 67 patients who underwent LPN or RAPN. The RNS and N-score were associated with CSE [6]. However, since the operative outcomes differ between LPN and RAPN, it is necessary to consider LPN and RAPN separately when analyzing the factors influencing CSE. Yoo et al. evaluated the impact of preoperative ureteral catheter insertion on urinary leakage after partial nephrectomy (PN). Ureteral catheter insertion was reported not to reduce the risk of urinary leakage after PN [7].

There have been reports concerning the prediction of postoperative complications of PN using the PADUA score and RNS [8, 9]. However, the factors associated with CSE in RAPN have not been reported. We evaluated the relationship between CSE and RNS in 101 cases of RAPN in the present study and found significant differences between the two groups regarding the R-score, E-score, N-score, RNS total score, and RNS total score excluding the L-score. Furthermore, CSE showed a moderate correlation with the RNS total score excluding the L-score (AUC 0.848, cut-off 5, sensitivity 0.83, specificity 0.73).

When the cut-off value for the RNS total score excluding the L-score was set to 4, the specificity and the sensitivity were 92% and 46%, respectively. CSE is thus considered unlikely to occur when the cut-off value is ≤ 4 points. These findings suggest that a ureteral catheter should not be placed in a patient with the RNS total score (excluding the L-score) of ≤ 4. Eliminating needless ureteral catheter indwelling reduces both the operative time and cost.

Several limitations associated with the present study warrant mention. First, this was a retrospective study. Second, the number of included patients was small. Future studies involving a larger cohort will be required. Finally, a small entry site may not have been found because the ureteral catheter could not be inserted in all cases. Even if there is no ureteral catheter placement, it is possible to identify the entry site (excluding micro entry sites) by improving the visualization by robot surgery. We also consider micro entry sites to not be clinically important. The purpose of ureteral catheter placement is precise closure rather than the identification of the entry sites.

## Conclusion

The RNS total score excluding the L-score was associated with CSE in our study. A ureteral catheter should not be placed in a patient with the RNS total score (excluding the L-score) of ≤ 4.

## Data Availability

All data and materials are available within the manuscript.

## References

[1] Ficarra V (2012). Eur Urol.

[2] Tamaszewski JJ (2014). Eur Urol.

[3] Bruner B (2011). BJU Int.

[4] Haber GP (2006). Eur Urol.

[5] Zargar H (2014). Int Braz J Urol..

[6] Kutikov A (2009). J Urol.

[7] Yoo S (2017). Clin Genitourin Cancer.

[8] Ficarra V (2009). Eur Urol.

[9] Schiavina R (2017). BJU int.

